# Address, before the Cincinnati Dental College Society

**Published:** 1865-12

**Authors:** WM. Taft


### ADDRESS.

BY WM. TAFT.

[Before the Cincinnati Dental College Society.]

GENTLEMEN: — At a meeting held in this room on last Saturday evening, for the purpose of organizing a Dental College Society, it was the expressed desire, that to-day, some one should lay before those assembled, the objects and purposes of the association we are forming. This seeming duty fell upon me, as your presiding officer; but, not without regret on my part, as there are others among you, abler, to clearly define the course which this society is intended to pursue. All the explanation, it seems to me necessary, would be the reading of article second of our constitution, which gives a concise and comprehensive explanation, one that could not be misunderstood by any.

However, we propose to go a little farther than mutual improvement, and professional intercourse. It is the design to build a structure that shall not fall at the close of the winter; one that shall be permanent and lasting, and live as long as there shall be a vestige of the Ohio College of Dental Surgery remaining. And, in future years, if our efforts shall be successful, we can look back to this time, and follow up the growth of large and influential associations, feeling a pride that we were among its founders.

By means of associated effort, the dental profession has advanced from a disgraced eatch-penny *trade*, to a position of honor and usefulness, inferior to no other profession that exists, und it is so respected, that a dentist can, unblushingly and without any sense of disgrace, tell his occupation. Why, I can, in my short life, recollect when I was almost ashamed to tell people, that my *father* was a dentist, when asked. To the rapid progress of the profession, and its high place, we owe to those noble and true hearted men, who have unflinchingly stood up in the infancy of the profession, battling in disgrace popular prejudice, that they might leave to us as a legacy, an honorable, and I think, the noblest profession that exists on the face of the earth; and, it is our duty to push forward the standard which they are leaving in our hands, until we can pass it as far again in the advance, from this time, as they have brought it to us.

So far as my knowledge reaches, we are pioneers in the formation of Dental College Societies of this kind. I believe we have organized the first one in the United States. The oldest dental society in the world, the Mississippi Valley Dental Association, meets here in this building, and why should we not have the oldest Dental College Society, meeting in the same place. As we have no precedent by which we can be governed, of course we must expect to encounter difficulties, but as we triumphantly meet them, we gain in strength. We have already found obstructions in our path, but we have overcome them, and our foundation is laid, which with the co-operation of a larger number than took part at the meeting Saturday night, we can make stronger and surer, then we shall move along harmoniously, to success. The small number at the first meeting was discouraging, but we recollected, that a few earnest workers could accomplish much.

The American Dental Association, the *largest* dental body in existence, was organized by eight or ten men. They struggled against almost every obstacle, and discouragement that could be thrown in their way. They looked upon their cause as nearly hopeless, but with hearts determined and right they succeeded. Every year has increased their members, giving them power and influence, new life to their energies, until, at last, a representative body, large and strong, possessing as much intelligence and genius, as respected and respectable as any society, in any profession. They have been welcomed to the embrace of the mother profession, medicine, where so long they have been a reproach, and a scorn, and recognized as a child, illused; but it was not a voluntary approach, the dental profession grew so strong, and large, that it could stand in argument with, and wear the clothes of the parent, so it was a forced recognition, and offer of her sympathies.

There are some in the dental profession that have fought and still fight associations. They are not true dentists, but what might be considered hangers on. They, blinded by their insatiate desires of pecuniary gain, can see no progress resulting from dental societies, but all the advancement they themselves make, they derive from published proceedings — they pretend not to see that either. There are some that say they cannot attend societies, because it costs money, well, they are to be pitied. It is an impossibility, for a dentist with any mind at all, to attend a dental society, without gaining five times the amount of knowledge, that his money is worth to him. For every dollar a man spends in attending, and assisting dental associations, and professional advancement, *will*, through the benefit he receives, throw one hundred dollars into his treasury. Now, these ignorant, miserable *wretches* that oppose dental societies, should be enlightened, and I think this society can do a good work in that direction. Gentlemen, our time is short, we have considerable work before us. I hope you will all take an interest, and active part in the society — contribute all you can to perpetuate our endeavors, and I am sure we will accomplish good to ourselves, our alma mater, and all with whom we may professionally come in contact.

